# Coronary artery calcification score as a prognostic indicator for COVID-19 mortality: evidence from a retrospective cohort study in Iran

**DOI:** 10.1097/MS9.0000000000001661

**Published:** 2024-04-04

**Authors:** Mohammad Taghi Hedayati Goudarzi, Saeed Abrotan, Naghmeh Ziaie, Kamyar Amin, Mehrdad Saravi, Seyed farzad Jalali, Roghayeh Pourkia, Iraj Jafaripour, Amir Moradi, Saeed kargar-soleimanabad, Homina Saffar

**Affiliations:** a Department of Cardiology, School of Medicine, Rouhani Hospital, Babol University of Medical Sciences, Babol; b Atherosclerosis Research Center, Ahvaz Jundishapur University of Medical Sciences, Ahvaz; c Student Research Committee, Faculty of Medicine, Mazandaran University of Medical Sciences, Sari, Iran

**Keywords:** cardiovascular disease, coronary artery calcification score, coronary artery calcification, coronavirus disease 2019, mortality

## Abstract

**Background:**

Coronary artery calcification (CAC) has been established as an independent risk factor for major adverse cardiovascular events. Nevertheless, the effect of CAC on in-hospital mortality and adverse clinical outcomes in patients with COVID-19 has yet to be determined.

**Objective:**

To investigate the association between CAC score and in-hospital mortality of COVID-19 patients

**Method:**

This retrospective cohort study was conducted across tertiary hospitals of University of Medical Sciences in Babol, a northern city in Iran, and enroled 551 confirmed COVID-19 patients with definitive clinical outcomes of death or discharge between March and October 2021. Demographic and clinical data, along with chest computed tomography (CT) findings and CAC score on admission, were systematically collected. The study utilized logistic regression analysis and Kaplan-Meier plots to explore the association between CAC score and in-hospital death and adverse clinical outcomes.

**Results:**

The mean age was 60.05±12.8. A significant difference regarding CAC score, age, history of hypertension, hyperlipidemia, cardiovascular diseases, and respiratory diseases among survivors and non-survivors was observed; however, gender was not found to be different. Furthermore, in multivariate analysis, CAC score greater than or equal to 400 [odds ratio (OR): 4.2, 95% CI: 1.70–10.33, *P* value: 0.002], hospitalization time (OR: 1.31, 95% CI: 1.13–1.53, *P* value < 0.001), length of ICU stay (OR: 2.02, 95% CI: 1.47–2.77, *P* value < 0.001), severe or critical COVID-19 severity in time of admission (95% CI: 1.79–18.29, *P* value: 0.003), and history of respiratory diseases (95% CI: 2.18–40, *P* value: 0.003) were found to be associated with higher odds of in-hospital mortality. Log-rank test also revealed a significant difference regarding the time of admission to death between patients with CAC score greater than or equal to 400 and those with CAC score less than 400 (*P* value < 0.001).

**Conclusion:**

Elevated CAC score is a crucial risk factor linked to in-hospital mortality and unfavourable clinical results in confirmed COVID-19 patients. This finding emphasizes the need for careful monitoring of individuals with high CAC scores.

## Introduction

Highlights

- Coronary artery calcification (CAC) is known as an independent risk factor for major adverse cardiovascular events.
- Elevated CAC score is a crucial risk factor linked to in-hospital mortality.
- Elevated CAC score is associated with unfavourable clinical results in COVID-19 patients.
- Careful monitoring of individuals with high CAC scores is Necessary.

The emergence of SARS-CoV-2 in 2019 has resulted in the COVID-19 pandemic, causing a wide range of symptoms, from asymptomatic to severe respiratory distress syndrome, pneumonia, and even death^1^. Notably, COVID-19 has been associated with various cardiac complications, including myocardial damage, acute heart failure, shock, and arrhythmia^2–4^. Studies have shown that~30% of patients with COVID-19 experience cardiac complications^5^, with a higher risk observed in those with a history of cardiovascular disease^6^. The SARS-CoV-2 virus enters its host cell by binding to the membrane receptor of angiotensin-converting enzyme 2 (ACE2)^7^, which is also expressed in the heart^8^. Consequently, COVID-19-related cardiac complications are prevalent, and potential associations between clinical outcomes of COVID-19 and cardiovascular risk factors require thorough investigation.

Coronary heart disease (CHD) has been proposed as a contributing factor to increased mortality in COVID-19^9^, and CAC has been identified as a potential measure of CHD^10^. CAC score, assessed by various scoring systems, including the Agatston score and volume score, has proven to be a reliable and quantitative predictor of cardiovascular events and mortality^11,12^. Its high sensitivity and negative predictive value make it a promising method for estimating the risk of cardiovascular disease, and it could potentially be used as a standalone prognostic tool^13^. Previous studies have demonstrated the utility of CAC scoring in risk stratification, with Agatston score in particular showing a strong correlation with adverse cardiovascular outcomes^14^. Recent research has shown that the presence of CAC, as assessed by chest computed tomography (CT) scan, in hospitalized COVID-19 patients is associated with an unfavourable prognosis^15^. CAC has also been linked to increased ventilation requirements, independent of age, and atherosclerotic heart disease^16^. However, further investigations are needed to establish definitive conclusions in this regard.

While some studies have identified relationships between cardiovascular diseases and COVID-19, the associations between COVID-19 clinical outcomes and risk factors for cardiovascular diseases, such as CAC score and its measuring techniques, are still being explored. Therefore, we aimed to investigate potential links between CAC score, history of previous diseases, hospital and ICU length of stay, COVID-19 severity, and in-hospital outcomes in order to shed light on this important area of research.

## Method

### Study design

This retrospective cohort was conducted at the teaching hospitals of University of Medical Sciences, from March to October 2021. The research was conducted in accordance with the ethical guidelines and was approved by the research ethics committee of University of Medical Sciences. The current study has been reported in line with the STROCSS criteria^17^.

### Patients and participant

A total of 551 confirmed COVID-19 patients, who were diagnosed with either a polymerase chain reaction test or chest CT scan and had at least moderate COVID-19 severity requiring hospitalization, were included in the study. Written informed consent was obtained from all subjects, and their personal information was kept confidential during data collection, transfer, and storage.

### Inclusion and exclusion criteria

Patient older than 18 and who has given an informed consent to participate in the study was included. Patients with the following conditions were excluded (1) pervious history of diseases that can interfere ca deposition, (2) history of myocardial infarction, (3) connective tissue diseases including Ehlers-Danus, Marfan’s syndrome.

### Data collection

Data on demographic characteristics, such as age, sex, duration of hospitalization, length of ICU stay, need for ventilation, COVID-19 symptoms, and history of diseases, were extracted from patients’ medical records and recorded in a checklist. In this study, to avoid bias in coding the data, one person initially coded all the information, and then another colleague checked the coding.

### Ca score evaluation method and instrument

CAC score was assessed by a radiologist using a chest CT scan (Siemens emotion 16 slice, Germany) and calculated by the Agatston scoring system. COVID-19 severity was defined according to the National Institute of Health guidelines^18^.

### Statistical analysis

Data were described by frequency for qualitative and mean and standard deviation for quantitative variables. The normality of data was tested using Kolmogrov–Smirnof normality test, and in case of normally distributed data, independent samples *t*-test and χ^2^ test, and in case of lack of normality of the data, their non-parametric counterparts were utilized. Additionally, logistic regression analysis was performed to identify independent risk factors of mortality among study participants. Data analysis was performed using SPSS software version 25 software, and a *P* value of less than 0.05 was considered statistically significant.

## Results

A total of 551 patients were included in the analysis, with a mean age of 60.05±12.8 years and a majority of male participants (Table 1).

**Table 1:** Categorized characteristics of study participants based on coronary artery calcification score

Variables	CAC score < 400 (*N*=466)	CAC score ≥ 400 (*N*=85)	*P*
Age (mean ± SD)	57.3±11.6	75.0±8.1	< 0.001
Calcium score (mean ± SD)	142.5±111.9	849.2±473.2	< 0.001
Hospitalization time (mean ± SD)	6.4±3.6	9.9±3.5	< 0.001
Days at ICU (mean ± SD)	1.0±1.7	4.3±1.6	< 0.001
Sex			
Female	223	40	0.893
Male	243	45	
COVID-19 severity at time of admission			
Moderate	318	10	< 0.001
Severe or critical	148	75	
Need for ventilation	67	69	< 0.001
History of myocardial infarction	81	42	< 0.001
History of cancer	5	7	< 0.001
History of respiratory diseases	5	9	< 0.001
History of hyperlipidemia	189	45	0.034
History of hypertension	124	50	< 0.001

CAC, coronary artery calcification.

Significant differences were observed between survivors and non-survivors with respect to various factors, including age, CAC score, hospitalization time, days spent in the ICU, requirement for ventilation, history of cardiovascular diseases, respiratory diseases, cancer, hyperlipidemia, and hypertension (all *P* values < 0.05). However, no significant association was found between sex and mortality (*P* value: 0.78). Notably, significant differences were also observed between patients with CAC score less than 400 and those with CAC score greater than or equal to 400 regarding the aforementioned factors, with no significant sex differences (*P* value: 0.89) (Table 2).

**Table 2:** Categorized characteristics of study participants base on survival

Variables	Survivors (*N*=486)	Non-survivors (*N*=65)	*P*
Age (mean ± SD)	58.6 ± 12.3	71.0 ± 11.5	< 0.001
CAC score (mean ± SD)	190.2 ± 237.6	710.3 ± 525.1	< 0.001
Hospitalization time (mean±SD)	6.6 ± 3.7	9.2 ± 3.5	< 0.001
Days at ICU (mean ± SD)	1.2 ± 1.8	4.2 ± 1.3	< 0.001
Sex			
Female	233	253	0.786
Male	30	35	
COVID-19 severity at time of admission			
Moderate	323	5	< 0.001
Severe or critical	163	60	
Need for ventilation	71	65	< 0.001
History of myocardial infarction	97	26	< 0.001
History of cancer	5	7	< 0.001
History of respiratory diseases	4	10	< 0.001
History of hyperlipidemia	197	37	0.012
History of hypertension	144	30	0.007

CAC, coronary artery calcification.

Univariate analysis revealed that mortality was significantly associated with age (*P* value < 0.001), CAC score equal to or greater than 400 (*P* value < 0.001), hospitalization time (*P* value < 0.001), days spent in the ICU (*P* value < 0.001), severe or critical COVID-19 severity at admission (*P* value < 0.001), history of cardiovascular diseases (*P* value < 0.001), respiratory diseases (*P* value < 0.001), cancer (*P* value < 0.001), hyperlipidemia (*P* value: 0.01), and hypertension (*P* value: 0.008) (Table 3).

**Table 3:** Univariate regression analysis of variables’ association with mortality

		95% CI		
Variables	OR	Lower	Upper	*P*
Age	1.088	1.061	1.116	< 0.001
Sex	0.931	0.554	1.564	0.786
CAC score ≥ 400	22.741	12.352	41.869	< 0.001
Hospitalization time	1.189	1.109	1.275	< 0.001
Days at ICU	2.036	1.739	2.386	< 0.001
COVID-19 severity at time of admission				
Moderate	Reference	—	—	—
Severe or critical	23.779	9.368	60.362	< 0.001
History of myocardial infarction	2.674	1.552	4.605	< 0.001
History of respiratory diseases	21.909	6.648	72.205	< 0.001
History of cancer	11.610	3.569	37.767	< 0.001
History of hyperlipidemia	1.939	1.149	3.271	0.013
History of hypertension	2.036	1.204	3.442	0.008

CAC, coronary artery calcification; OR, odds ratio.

Multivariate analysis further revealed that patients with a CAC score equal to or greater than 400 had four times higher odds of mortality compared with those who had CAC score of less than 400 (95% CI: 1.70–10.33, *P* value: 0.002). Moreover, longer hospitalization time [odds ratio (OR): 1.31, 95% CI: 1.13–1.53, *P* value < 0.001] and longer ICU stays (OR: 2.02, 95% CI: 1.47–2.77, *P* value < 0.001) were also associated with increased odds of death. Furthermore, patients with severe or critical COVID-19 at admission had 5.7-fold higher odds of mortality compared to those with moderate COVID-19 severity (95% CI: 1.79–18.29, *P* value: 0.003), while hospitalized COVID-19 patients with a history of respiratory diseases were 9.3 times more likely to die than those without such a history (95% CI: 2.18–40, *P* value: 0.003) (Table 4).

**Table 4:** Multivariate regression analysis of variables’ association with mortality

		95% CI		
Variables	OR	Lower	Upper	*P*
Age	1.02	0.96	1.03	0.857
Calcium score ≥ 400	4.202	1.708	10.336	0.002
Hospitalization time	1.31	1.132	1.535	< 0.001
Days at ICU	2.02	1.477	2.777	< 0.001
COVID-19 severity at time of admission				
Moderate	Reference	—	—	—
Severe or critical	5.737	1.799	18.297	0.003
History of myocardial infarction	1.184	0.544	2.575	0.671
History of cancer	4.424	0.695	27.777	0.115
History of respiratory diseases	9.345	2.183	40	0.003
History of hyperlipidemia	1.428	0.702	2.906	0.324
History of hypertension	1.011	0.471	2.169	0.979

CAC, coronary artery calcification; OR, odds ratio.

**Figure 1:**
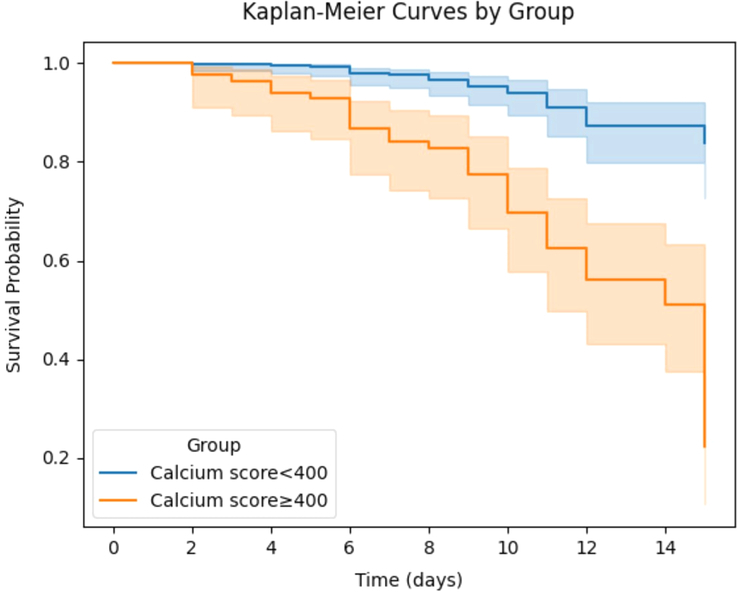
Kaplan–Meier curve for in-hospital mortality between low and high coronary artery calcification (CAC) groups. Patients with high CAC score had a higher mortality rate in log-rank test (χ^2^: 42.312, *P* value < 0.001).

Finally, a log-rank test was performed to compare the time from admission to death between patients with a CAC score equal to or greater than 400 and those with a score less than 400, revealing a significant difference between the two groups (χ^2^: 42.312, *P* value < 0.001) (Fig. 1).

## Discussion

The present retrospective cohort study aimed to investigate the clinical factors associated with mortality among hospitalized patients with COVID-19 at our institution. Our analysis of 551 patients revealed that age, CAC score, hospitalization time, days spent in the ICU, requirement for ventilation, and comorbidities such as cardiovascular diseases, respiratory diseases, cancer, hyperlipidemia, and hypertension were significantly associated with mortality. Furthermore, a multivariate analysis identified CAC score greater than or equal to 400, longer hospitalization time, longer ICU stays, severe or critical COVID-19 at admission, and a history of respiratory diseases as independent risk factors for mortality.

The primary cause of death in COVID-19 infection is commonly attributed to respiratory failure. However, there is evidence that cardiac manifestations may also contribute to overall mortality and can even be the primary cause of death^19^. The CAC score has been shown to be a reliable and quantitative predictor utilizing various scoring systems such as Agatston score and volume score^12,20^. CAC score can be readily assessed on a routinely performed CT scan of the chest, which is currently routinely performed in nearly all admitted COVID-19 patients for evaluation of pulmonary disease severity and the extent of lung dysfunction^21–25^. In a study conducted by Giannini *et al*.^26^, it was observed that non-survivor COVID-19 patients had significantly higher CAC scores (467±570 vs. 206±424), and CAC scores were found to be an independent predictor of mortality. In addition, Fovino *et al*.^27^ also reported a significant difference regarding mortality rate and between COVID-19 patients with CAC score greater than or equal to 400 and those with CAC score less than 400. Similarly, Gupta *et al*.^28^ observed a positive correlation between higher CAC scores and increased mortality rates and identified severe coronary calcification as an independent predictor for mortality. In line with the current literature, Our study also observed a significant difference in the CAC score between COVID-19 patients who deceased in the hospital compared with survivors, and CAC scores equal to or higher than 400 appeared to be an independent risk factor for in-hospital mortality. The findings can be explained by several mechanisms. One possible explanation would be that patients with high CAC scores may have pre-existing cardiovascular disease that predisposes them to severe COVID-19 and unfavourable outcomes. Moreover, atherosclerosis is known to induce endothelial dysfunction, which, when combined with COVID-19 infection, may potentiate viral replication and subsequently trigger heightened inflammatory responses, thereby exacerbating pre-existing cardiovascular disease and resulting in worse clinical outcomes. These observations underscore the importance of CAC score assessment in identifying individuals at high risk for adverse outcomes.

In our study, we also observed a significant association between high CAC score and hospitalization or ICU length of stay in COVID-19 patients. Moreover, our findings indicated that longer hospitalization or ICU stays were linked to higher mortality rates in COVID-19 patients. These findings are consistent with previous studies that reported that COVID-19 patients who died had longer hospitalization stays or ICU admission compared with survivors^29–32^. As more severe forms of COVID-19 are associated with higher mortality rates, patients with severe forms of COVID-19 may need longer in-hospital care or ICU admission. Additionally, hospital-acquired pneumonia rates can increase with longer in-hospital stays and exacerbate pre-existing COVID-19 severity, ultimately impacting patients’ outcomes.

The association between CAC score and ICU admission in COVID-19 patients remains controversial. Fovino *et al*.^27^ found a significant difference in ICU admissions between patients with CAC scores less than 400 and those with CAC scores greater than or equal to 400. Similarly, Zimmerman *et al*.^33^ reported that ICU admission and longer ICU stay were associated with higher odds of mortality in COVID-19 patients with CAC scores above the median. However, some studies have not reported a significant association between CAC score and ICU admission or length of ICU stay^32,34,35^. This variability in findings could be attributed to differences in patient categorization based on CAC score, sample size, or CAC scoring method. For example, Pergola and colleagues categorized patients into those with a CAC score of zero and those with a CAC score of greater than or equal to 1. Additionally, Mousseaux *et al*.^35^ utilized a visual scoring system instead of the Agatston scoring method used in the current study.

Our study revealed significant associations between mortality and comorbidities, including cardiovascular diseases, respiratory diseases, cancer, hyperlipidemia, and hypertension. Of note, a history of respiratory diseases was identified as an independent predictor of mortality among COVID-19 patients. These findings are in line with previous studies that have demonstrated the adverse impact of comorbidities on COVID-19 outcomes^36–38^, consistent with evidence reported by Wu *et al*.^39^ who conducted a meta-analysis that indicated a 3.5-fold increase in the odds of mortality for COVID-19 patients with chronic respiratory diseases. Moreover, some studies have identified chronic obstructive pulmonary disease as an independent risk factor for predicting mortality among COVID-19 patients^40,41^.

In terms of age, our study revealed a significant difference in age between survivors and non-survivors, as well as patients with CAC score greater than or equal to 400 and patients with CAC score less than 400; however, age was not identified as an independent risk factor for mortality. Similarly, Gupta *et al*.^28^ did not find age to be an independent predictor of death in COVID-19 patients. In contrast, many studies have recognized age as an independent predictor of death in COVID-19 patients^32,34,35,42,43^. This disparity can be attributed to differences in study populations, as our patients had a lower mean age compared to previous studies.

Furthermore, our study did not observe any correlation between sex and the odds of death. The consideration of sex as an independent predicting factor for death in COVID-19 patients remains controversial among researchers and clinicians. While some studies did not recognize sex as a risk factor for mortality^28,32,34,43,44^, others identified male sex as associated with higher odds of mortality in COVID-19 patients^26,42,45^. It is apparent that larger studies are necessary to reach a definite conclusion regarding the effects of sex differences on adverse outcomes in COVID-19 patients.

## Conclusion

In conclusion, our study revealed that a CAC score equal to or greater than 400, longer hospitalization time and ICU stays, and a history of respiratory diseases are independent predictors of mortality in COVID-19 patients. Notably, the assessment of CAC score can be conveniently performed during COVID-19 patients’ lung CT scan, offering a feasible approach to identify high-risk patients for targeted management strategies. Such findings emphasize the importance of incorporating CAC score assessment into the clinical evaluation of COVID-19 patients to improve risk stratification and management outcomes.

### Limitations

One limitation of our study is its retrospective design, which may limit the ability to establish causality and generalize our findings to other populations. Additionally, our study was conducted at a single institution, which may limit the generalizability of our findings to other settings. Future studies with larger sample sizes and more diverse patient populations are needed to confirm and expand upon our findings.

## Ethical approval

This retrospective cohort was conducted at the teaching hospitals of Babol University of Medical Sciences, Babol, Iran, from March to October 2021. The research was conducted in accordance with the ethical guidelines and was approved by the research ethics committee of Babol University of Medical Sciences (Ethics approval number IR.MUBABOL.REC.1399.394).

## Consent for publication

Written informed consent was obtained from the patients for publication and any accompanying images. Copies of the written consent are available for review by the Editor-in-Chief of this journal on request.

## Sources of funding

None.

## Author contribution

I.J., M.T., S.A., N.Z., K.A., M.S. and F.J. involved in interpretation and collecting of data, and editing the manuscript. R.P., A.M., S.K. and H.S. involved in writing, editing and preparing the final version of manuscript. All authors reviewed the paper and approved the final version of the manuscript.

## Conflicts of interest disclosure

The authors declared no conflicts of interest.

## Research registration unique identifying number (UIN)

1. Name of the registry: researchregistry Unique identifying number or registration ID: : researchregistry9365.
2. Hyperlink to your specific registration (must be publicly accessible and will be checked): https://www.researchregistry.com/browse-theregistry#home/registrationdetails/64cba34ed338670029b7009c/.

## Guarantor

Iraj Jafaripour.

## Availability of data and materials

Data and materials are available from the corresponding author on reasonable request.

## Provenance and peer review

Current study isn’t invited.
